# Preparation, Characterization, and Antioxidant Activity of L-Ascorbic Acid/HP-*β*-Cyclodextrin Inclusion Complex-Incorporated Electrospun Nanofibers

**DOI:** 10.3390/foods12071363

**Published:** 2023-03-23

**Authors:** Nabab Khan, Amit Kumar Singh, Ankit Saneja

**Affiliations:** 1 Formulation Laboratory, Dietetics and Nutrition Technology Division, CSIR—Institute of Himalayan Bioresource Technology, Palampur 176061, India; 2 Academy of Scientific and Innovative Research (AcSIR), Ghaziabad 201002, India

**Keywords:** ascorbic acid, cyclodextrin, nanofibers, electrospinning, antioxidant

## Abstract

L-Ascorbic acid (LAA) is a key vitamin, implicated in a variety of physiological processes in humans. Due to its free radical scavenging activity, it is extensively employed as an excipient in pharmaceutical products and food supplements. However, its application is greatly impeded by poor thermal and aqueous stability. Herein, to improve the stability and inhibit oxidative degradation, we prepared LAA-cyclodextrin inclusion complex-incorporated nanofibers (NFs). The continuous variation method (Job plot) demonstrated that LAA forms inclusions with hydroxypropyl-*β*-cyclodextrin (HP-*β*-CD) at a 2:1 molar stoichiometric ratio. The NFs were prepared via the single step electrospinning technique, without using any polymer matrix. The solid-state characterizations of LAA/HP-*β*-CD-NF via powder x-ray diffractometry (PXRD), Fourier-transform infrared (FT-IR) analysis, differential scanning calorimetry (DSC), thermal gravimetric analysis (TGA), and nuclear magnetic resonance (^1^H NMR and 2D-NOESY) spectroscopy, reveal the effective encapsulation of the LAA (guest molecule) inside the HP-*β*-CD (host) cavity. The SEM micrograph reveals an average fiber diameter of ~339 nm. The outcomes of the thermal investigations demonstrated that encapsulation of LAA within HP-*β*-CD cavities provides improved thermal stability of LAA (by increasing the thermal degradation temperature). The radical scavenging assay demonstrated the enhanced antioxidant potential of LAA/HP-*β*-CD-NF, as compared to native LAA. Overall, the study shows that cyclodextrin inclusion complex-incorporated NFs, are an effective approach for improving the limitations associated with LAA, and provide promising avenues in its therapeutic and food applications.

## 1. Introduction

The vital component L-ascorbic acid (LAA), also known as vitamin C, is particularly common in foods and food supplements [1]. It plays a crucial part in the formation of collagen and acts as a radical scavenger in biological systems [2]. LAA has been extensively employed by the pharmaceutical, cosmetic, and food sectors, due to its bioactivity and antioxidant potential [3].

Despite several benefits of LAA as an excipient, it suffers from several limitations, such as, it is highly sensitive to environmental conditions and external effects (exposure to heat, light, moisture, oxygen, metal ions, and structural modifications), which hinders its incorporation into food supplements, cosmeceuticals, and pharmaceutical products [4,5]. Therefore, research is ongoing to protect LAA from thermal and oxidative degradation. Cyclodextrins (CD) and their modified forms, are widely applied in the food sector for encapsulation of active agents, because of their capability to form a host–guest inclusion assembly [6,7]. The molecular encapsulation of bioactive molecules and functional additives/excipients by CDs, improves thermal and oxidative stability, provides controlled release, prolongs shelf-life, enhances the bioavailability, and masks the bitter taste of bioactive molecules [8,9,10]. It has already been reported that the molecular encapsulation of bioactive entities in cyclodextrin cavities provides enhancements in the stability and shelf-life of the guest entity [11].

In recent years, electrospinning technology has gained tremendous attention and has been demonstrated to be a very promising approach for the encapsulation of bioactive molecules, in the food and pharmaceutical sectors [12,13,14,15,16]. The electrospinning technique is frequently used to create nanofibers (NFs) with distinctive qualities, such as a large surface to volume ratio, a nanosized architecture, a high porosity, and a light weight, enabling their application in food, pharmaceutical, and cosmeceutical products [17,18].

Different classes of bioactives (e.g., flavors, vitamins, essential oils, and food supplements, etc.) have been incorporated within NFs webs/mesh via the electrospinning technique, for food and pharmaceutical applications [19,20,21,22,23]. Furthermore, electrospun NFs can be produced with a high concentration CD solution, without using any polymer or organic solvents (polymer-free electrospun solution). In recent years, various studies have been reported where bioactive agents such as cinnamaldehyde, ferulic acid, resveratrol, etc., were encapsulated in different cyclodextrins, and the obtained polymer-free solution was utilized to form NFs [24,25,26].

This work describes our efforts to prepare stable LAA/cyclodextrin inclusion complex-integrated nanofibers for better LAA stability and enhanced antioxidant potential of LAA, using polymer-free electrospinning [20,21,26,27]. In this study, we have chosen a modified version of *β*-cyclodextrin, *viz*. hydroxypropyl-beta-cyclodextrin (HP-*β*-CD), for the inclusion of LAA. HP-*β*-CD has a highly hydrophilic nature, making the electrospinning of HP-*β*-CD-encapsulated bioactives a straight-forward process, without the need for any additional polymer matrix. Here, the molar stoichiometric ratio of guest and host was initially determined via constructing the Job plot. A clear transparent solution of LAA/HP-*β*-CD was prepared and incubated, to form an inclusion complex, and uniform LAA/HP-*β*-CD nanofibers were obtained via the electrospinning equipment. The obtained NFs were analyzed via powder X-ray diffractometry (PXRD), Fourier-transform infrared (FT-IR) analysis, differential scanning calorimetry (DSC), and thermal gravimetric analysis (TGA). The surface morphology was investigated through scanning electron microscopy (SEM). The inclusion of LAA inside the hollow cavity of HP-*β*-CD, and molecular interaction, was confirmed via nuclear magnetic resonance (NMR). Furthermore, the antioxidant potential of LAA/HP-*β*-CD-NF was also determined through a radical scavenging assay.

## 2. Materials and Methods

### 2.1. Materials

L-Ascorbic acid (C_6_H_8_O_6_, Mw: 176.13, ≥98% purity, CAS No.: 50-81-7) was purchased from MP Biomedicals. Hydroxypropyl-*β*-cyclodextrin (CAS No.: 128446-35-5) was purchased from Tokyo Chemical Industry Co., Ltd. (Tokyo, Japan). Deuterium oxide (“100%”, ≥99.96 atom %D, CAS No.: 7789-20-0), DMSO-d_6_ (“100%”, 99.96 atom %D, contains 0.03% (*v*/*v*) TMS, CAS No.: 2206-27-1), and 2,2-diphenyl-1-picrylhydrazyl (DPPH) (CAS No.: 1896-66-4) were purchased from Sigma-Aldrich (USA). All other chemicals and reagents used in this research work were of analytical grade unless otherwise stated.

### 2.2. Preparation of Electrospinning Solution

Briefly, HP-*β*-CD (200% *w*/*v*) concentrated solution was prepared in Milli-Q water. Afterwards, LAA was added to the clear HP-*β*-CD solution to obtain a 2:1 (LAA/HP-*β*-CD) molar ratio. The resulting aqueous solution was stirred at room temperature, in order to obtain a clear LAA/HP-*β*-CD solution [24].

### 2.3. Electrospinning of LAA/HP-β-CD Solution

**Figure 1:**
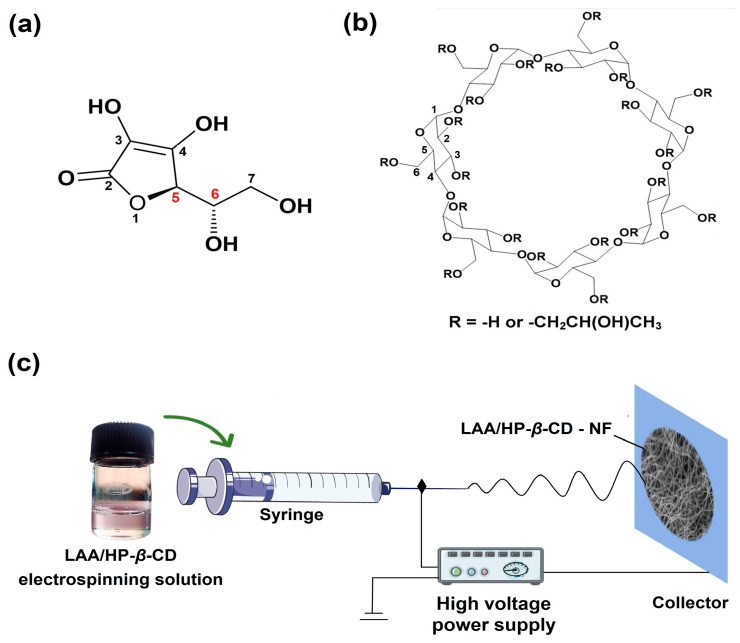
Schematic illustration of molecular structure of: (**a**) L-ascorbic acid, (**b**) hydroxypropyl-*β*-cyclodextrin, and (**c**) formation of LAA/HP-*β*-CD-NFs, via the electrospinning technique.

Electrospinning equipment (E-SPIN NANOTECH, model: Super ES-1, Kanpur, India) was used for the NF production. The clear LAA/HP-*β*-CD solution (2:1) was filled into 1 mL syringes and a metal needle (21 G) was attached over the filled syringes. During the electrospinning period, the LAA/HP-*β*-CD clear solution was passed from the syringes at a steady flow rate (0.5 mL/h). The roller collector was wrapped with aluminum foil and located 15 cm away from the needle tip. A constant high voltage (15 kV) was supplied during the electrospinning period (Figure 1). The NFs deposited over the roller collector were recovered [28,29]. The temperature inside the chamber was recorded as 20–25 °C (approx.) and humidity was recorded as ~50%, during the electrospinning period.

### 2.4. Characterization and Measurements

#### 2.4.1. Construction of Job Plot

To determine the molar stoichiometric ratio of the host–guest complex formation, the molar ratio of HP-*β*-CD and LAA was continually altered, without changing the total volume and total molar concentration [(LAA) + (HP-*β*-CD)]. The change in absorbance intensity (∆A) of LAA, in the absence (Abs_0_) and presence of HP-*β*-CD (Abs), was recorded using a UV-Visible spectrophotometer (Genesys 180, Thermo Scientific, Madison, WI, USA) at 261 nm, under corresponding conditions [30]. The Job plot was obtained by plotting ∆A*R against R. The value of R was calculated as follows:

(1) $$R=\left[\frac{\left[LAA\right]}{\left[\left(LAA\right)+\left(HP-\beta -CD\right)\right]}\right]$$

where [LAA] is the molar concentration of L-ascorbic acid and [(LAA) + (HP-*β*-CD)] is the total molar concentration of L-ascorbic acid and HP-*β*-CD in the solution.

#### 2.4.2. Determination of Conductivity and Viscosity of Electrospinning Solution, and Entrapment Efficiency of LAA/HP-*β*-CD-NF

The conductivity of the LAA/HP-*β*-CD solution was measured by using a conductivity meter (deluxe conductivity meter, Model-602), at 21 ± 1 °C. A viscometer (IKA^®^ ROTAVISC LO-VI S000, Staufen, Germany) was used to measure the viscosity of the LAA/HP-*β*-CD solution, at 23 ± 1 °C. The entrapment efficiency of LAA/HP-*β*-CD-NF was assessed by high-performance liquid chromatography (HPLC), as per the method described by Reibero et al., with minor modifications [31]. The quantitative analysis of LAA was performed utilizing an HPLC system (Agilent 1260 Infinity II, Waldbronn, Germany) equipped with a quaternary pump (G7111A, 1260 Quat pump VL), an automatic vial sampler injector (G7129A, 1260 Vialsampler), a multicolumn thermostat (G7116A, 1260 MCT), and a DAD detector (G7115A, 1260 DADWR). A reverse-phase C18 column (LiChrospher^®^ 100 RP-18 Hibar^®^ RT 250 mm × 4.6 mm, 5 µm, Darmstadt, Germany) was used to quantify LAA. The LAA content was evaluated using isocratic elution mode [eluent A: 100% phosphate buffer (pH 3.0); eluent B: 100% methanol (99/1, *v*/*v*)] at a flow rate of 1 mL/min at a specified wavelength.

(2) $$Entrapment\;efficiency=\left(\frac{practical\;amount\;of\;LAA\;in\;NF}{theoretical\;amount\;of\;LAA\;in\;NF}\right)\times 100$$

#### 2.4.3. Morphological Analysis of Nanofibers

A scanning electron microscope (SEM, Hitachi S-3400N, Tokyo, Japan) was employed to perform the morphological analysis of the LAA/HP-*β*-CD-NF. Prior to the analysis, a small piece of NF was attached over SEM stubs, using double-sided carbon adhesive tape. To make the samples electrically conductive, they were sputter coated with a thin film of gold/palladium (ion sputter coater, Hitachi-1030). SEM micrographs were captured from 10 mm working distance, at an accelerating voltage of 15 kV. Using the ImageJ software, the average NF diameter (AD, mean ± SD) was determined, based on at least 50 measurements, from different locations of micrographs [32].

#### 2.4.4. Fourier-Transform Infrared Spectroscopy (FT-IR) Analyses

An FT-IR spectrophotometer (PerkinElmer, installed with software spectrum ES version 10.5.3, Waltham, MA, USA) was employed to acquire the FT-IR spectra of LAA, HP-*β*-CD, and LAA/HP-*β*-CD-NF. Prior to the analysis, KBr pellets were prepared by crushing the samples with KBr and further pressing them with a hydraulic press. The spectrum was acquired using the optimum conditions, spectral range of 4000 cm^−1^ to 500 cm^−1^, and resolution of 4 cm^−1^.

#### 2.4.5. Powder X-ray Diffractometry (PXRD) Analyses

The X-ray diffraction patterns of LAA, HP-*β*-CD, and LAA/HP-*β*-CD-NF were investigated using an X-ray diffractometer (SmartLab 9 kW rotating anode, Rigaku Corporation, Tokyo, Japan) with a Cu-Kα radiation source installed. Prior to analysis, the samples were tightly sealed in a rectangular aluminum pan. The diffraction pattern was acquired in the range of 5–60° at 2θ diffraction angle, with a 10°/min scan rate and 0.02° step size.

#### 2.4.6. Thermal Behavior Analysis

The thermal properties of LAA, HP-*β*-CD, and LAA/HP-*β*-CD-NF were evaluated using a TGA/DSC thermal analyzer (Mettler Toledo, model: TGA/DSC-I, Columbus, OH, USA). The TGA thermograms were acquired from 25 °C to 700 °C, under a nitrogen atmosphere, at a heating rate of 10 °C/min. DSC thermograms were recorded simultaneously.

#### 2.4.7. Nuclear Magnetic Resonance (NMR) Analyses

To examine the stereochemical interaction between the host and guest molecules, ^1^H NMR spectra of the LAA, HP-*β*-CD, and LAA/HP-*β*-CD-NF, and 2D-NOESY spectra of LAA/HP-*β*-CD-NF, were acquired with the experimental conditions as follows: number of scans—256, acquisition time—0.170 s, and relaxation delay—2.0 s. The D_2_O/DMSO-d_6_ (600 µL) solvent was used to record the NMR spectra on an NMR spectrometer (AV-600, Bruker, Switzerland), at room temperature. The changes in chemical shifts were counted as parts per million (ppm).

#### 2.4.8. DPPH Antioxidant Radical Scavenging Assay

The free radical scavenging abilities of LAA and LAA/HP-*β*-CD-NF were examined using the 2,2-diphenyl-1-picrylhydrazyl (DPPH) free radical scavenging assay. Prior to the analysis, the pure LAA and LAA/HP-*β*-CD-NF (equivalent to pure LAA) were dissolved in methanol, with the increasing LAA concentration ranging from 10 µM to 30 µM. In 2 mL Eppendorf tubes, containing varying concentrations of LAA, methanolic solution of DPPH (100 μM) was added at a volume ratio of 1/1, *v*/*v* (DPPH/sample). The Eppendorf tubes were incubated at 37 °C, under dark conditions, for 30 min. The optical absorbance intensity of the samples was recorded at 517 nm, using a UV-Vis spectrophotometer (Genesys 180, UV spectrophotometer) [21]. By using the following equation, the % DPPH radical scavenging of each sample was determined:

(3) $$\%\;DPPH\;radical\;scavenging=\lfloor \frac{\left(Ac-As\right)\;}{Ac}\rfloor \times 100$$

where *As* is the absorbance intensity of samples containing LAA, and *Ac* is the absorbance intensity of the stock DPPH solution. The experiments were conducted in triplicate and data were expressed as mean ± standard deviation [26].

#### 2.4.9. Statistical Analyses

All replicated experimental results (n ≥ 3) were presented as mean values ± standard deviations. The statistical analysis, at the significance level of *p* ˂ 0.05, was achieved by using one-way analysis of variance (ANOVA) followed by Tukey’s post hoc test, using the GraphPad Prism 9 software.

## 3. Results and Discussion

### 3.1. Determination of Complex Stoichiometry via Job Plot

**Figure 2:**
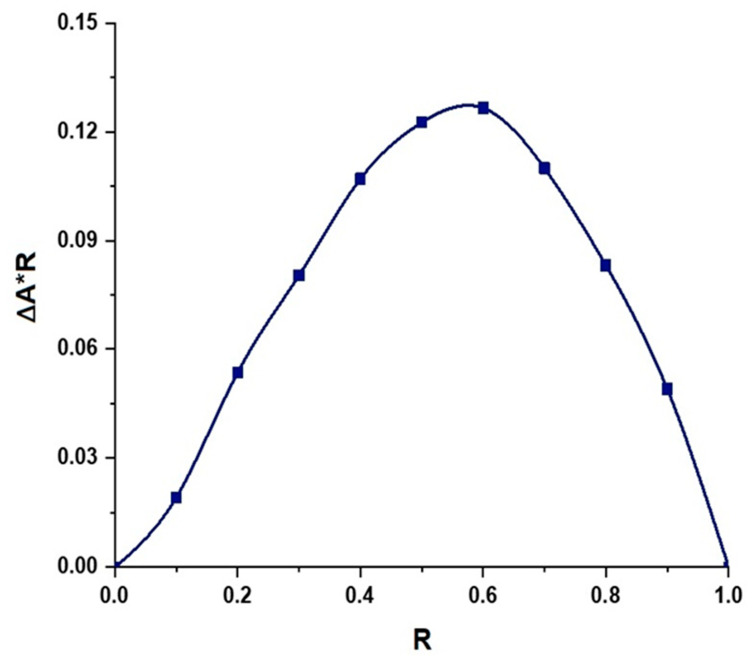
Graphical representation of continuous variation plot (Job plot) for the complexation of LAA with HP-*β*-CD, from absorbance measurements at 25 °C. R = [(LAA)/ (LAA) + (HP-*β*-CD)], ∆A = absorbance difference of LAA with and without HP-*β*-CD.

Job’s continuous variation method was employed for the determination of the molar stoichiometric ratio of the guest–host inclusion formation complex [11,33]. The representative Job plot curve of the LAA/HP-*β*-CD complex is presented in Figure 2. The value of R at the maximum deviation, provides the molar stoichiometric ratio of the guest–host required to form the inclusion complex. Saha et al. reported that the ratio of guest–host is 1:2 if the value of R = 0.33, 1:1 if R = 0.5, and 2:1 if R = 0.66 [11]. In the present study, the Job plot demonstrated an R value of ~0.63, which suggests a 2:1 stoichiometry of LAA with HP-*β*-CD (Supplementary Table S1) [34,35].

### 3.2. Measurement of Conductivity and Viscosity of the Electrospinning Solution, and Entrapment Efficiency of LAA/ HP-β-CD-NF

The conductivity and viscosity of the electrospinning solution are two crucial parameters, that have a direct impact on the morphology of the LAA/ HP-*β*-CD-NF. The conductivity and viscosity of the electrospinning solution were also determined as part of this study. The conductivity and viscosity of the 2:1 molar stoichiometric ratio LAA/HP-*β*-CD solution, were determined to be 80 µS/cm and 2.252 ± 0.0149 Pa·s, respectively [26,36]. The efficiency of entrapment of the LAA into LAA/HP-*β*-CD-NF depends on the solubility of LAA in the LAA/HP-*β*-CD solution, and the effective incorporation of LAA in the cavity of HP-*β*-CD. The average percentage entrapment efficiency of the obtained LAA/HP-*β*-CD-NF was found to be 97.30 ± 0.55%, which indicated that the electrospun LAA/HP-*β*-CD-NF nanofibers had a high entrapment efficiency, with negligible loss in LAA during nanofiber formation [37].

### 3.3. Surface Morphology of Nanofibers

**Figure 3:**
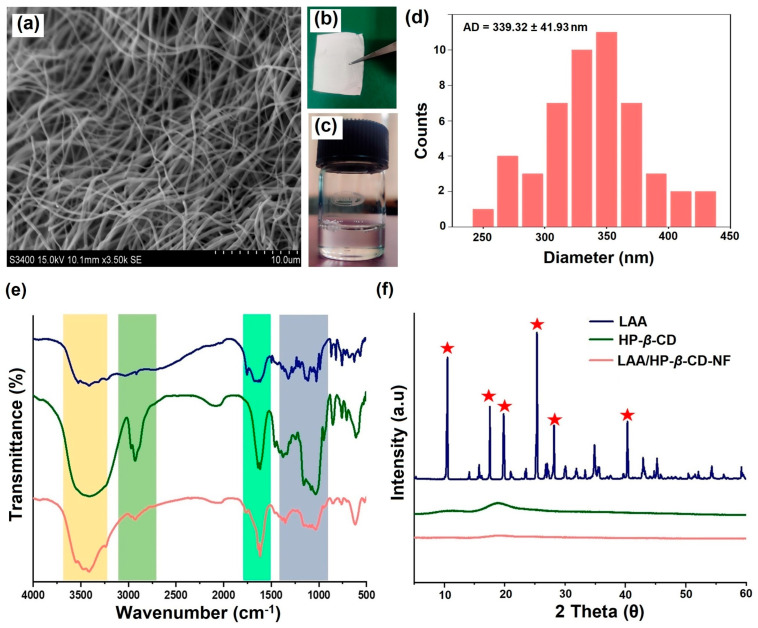
Solid state characterization of LAA/HP-*β*-CD-NFs. (**a**) Surface micrograph of LAA/HP-*β*-CD-NFs captured by scanning electron microscope, (**b**,**c**) photographs of the obtained LAA/HP-*β*-CD-NFs and electrospinning solution, respectively, (**d**) histogram representing average diameter of LAA/HP-*β*-CD-NFs, determined using the ImageJ 1.53t software, based on at least 50 counts, (**e**) FT-IR spectrum and (**f**) XRD diffractogram of LAA (blue, stars in red color indicates sharp diffraction peaks), HP-*β*-CD (green), and LAA/HP-*β*-CD-NFs (orange).

The surface morphology of the fabricated LAA/ HP-*β*-CD-NF displayed a randomly aligned morphology, without any indication of beads (Figure 3a). Nonetheless, there were variations in the diameter of the produced NFs. The mean diameter of the LAA/HP-*β*-CD-NF, evaluated using the ImageJ 1.53t software, demonstrated an average diameter value of 339.32 ± 41.93 nm (Figure 3d). The micrographs showed that, under the optimized electrospinning parameters (including voltage, distance, flow rate), NFs without the presence of any beads/particles can be produced. Furthermore, the diameter and morphology of the obtained NFs were similar to previously published reports [27,36]. For instance, Celebioglu et al. reported that an electrospinning solution with a lower viscosity and higher conductivity, generates fine fibers, due to the stretching of the jet during the electrospinning process. In addition, the study also reported an average diameter of 220 ± 70 nm for the developed ferulic acid/HP-*β*-CD-incorporated nanomats [25].

### 3.4. Fourier-Transform Infrared Spectroscopy (FT-IR) Studies

We investigated the interaction between LAA and HP-*β*-CD using FT-IR spectroscopy. The FT-IR spectrum of LAA demonstrated multiple peaks, at 3524.86 cm^−1^, 3407.55 cm^−1^, and 3314.40 cm^−1^ due to stretching of different hydroxyl groups. The peaks at 3031.48 cm^−1^ and 2914.17 cm^−1^ were attributed to C–H stretching. The C=O stretching of the five-membered lactone ring and the C=C ring stretching, were responsible for the absorption bands that occurred at 1754.89 cm^−1^ and 1672.09 cm^−1^, respectively. The peak at 1320.16 cm^−1^ was due to an enol hydroxyl group stretch, all these observations were similar to the previously published report by Ahmed et al. (Figure 3e) [38,39]. The FT-IR spectrum of HP-*β*-CD revealed a characteristic absorption band at 3414.45 cm^−1^, a result of the hydrogen-bonded hydroxyl groups experiencing symmetrical and asymmetrical stretching vibration. The C–H bonds’ stretching vibration was assigned a peak at 2931.42 cm^−1^, while other notable bands at 1644.48 cm^−1^, 1161.45 cm^−1^, and 1033.79 cm^−1^, represent H–O–H bending, C–O stretching vibration, and C-O-C vibration, respectively [40]. The absorption bands of LAA were covered by intense bands of HP-*β*-CD in the case of LAA/HP-*β*-CD-NF, largely as a result of the increased content of HP-*β*-CD in the NF samples, and the full range FT-IR spectrum of LAA/HP-*β*-CD-NF was very similar to the pure HP-*β*-CD spectrum. In contrast, some LAA peaks were visible in the LAA/HP-*β*-CD-NF spectrum, with a skewed and attenuated profile that differs from the pure LAA spectrum (highlighted with various shades in Figure 3e). Similar conclusions have been published in previous reports, where the characteristic absorption bands of the guest molecule were moved, diminished, or obscured, as a result of the guest molecule’s insertion inside the host cavity [25,40]. The observable discrepancies between the FT-IR spectra of the complex state and the pristine LAA, provide a good indication of the potential molecular interactions between LAA and HP-*β*-CD cavities. Further analysis was carried out, to establish the incorporation of LAA inside HP-*β*-CD cavities.

### 3.5. Powder X-ray Diffractometry (PXRD) Analysis

PXRD analysis is a versatile technique to investigate the physical state (amorphous/crystalline) of LAA incorporated in NF. The LAA diffractogram demonstrated several significant sharp peaks at different diffraction angles: 10.5°, 17.5°, 19.8°, 25.3°, 28.1°, and 40.3°, due to its existence in crystalline form (Figure 3f) [41]. By contrast, pristine HP-*β*-CD displayed a smooth-patterned dull halo curve, confirming its amorphous state, which was similar to previously published reports [25,40]. Conversely, the PXRD pattern of the LAA/HP-*β*-CD-NF, demonstrated an amorphous diffractogram, being identical to the diffractogram of HP-*β*-CD, with complete disappearance of the characteristics of the LAA crystal diffraction patterns, suggesting the effective incorporation of LAA inside the HP-*β*-CD cavities.

### 3.6. Thermal Behavior Analyses

**Figure 4:**
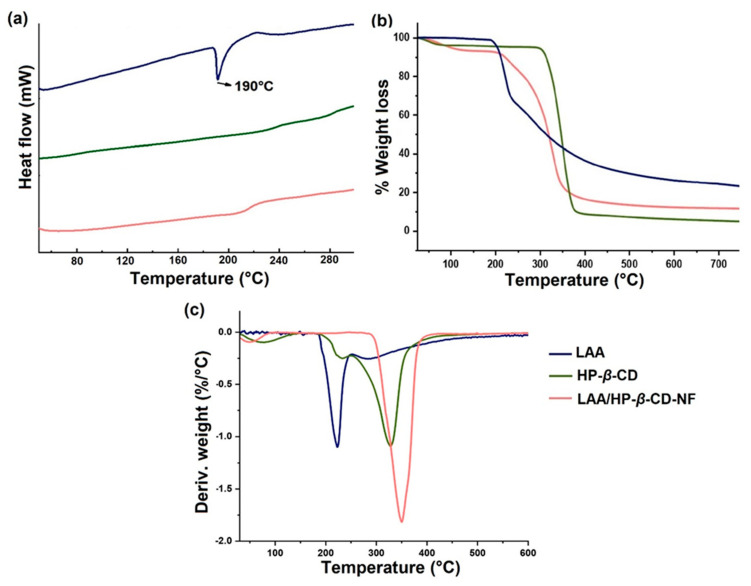
Thermal analysis representing overlay thermogram: (**a**) DSC, (**b**) TGA, and (**c**) DTG analyses of LAA (blue), HP-*β*-CD (green), and LAA/HP-*β*-CD-NFs (orange).

The thermal properties of LAA incorporated in NFs, were studied by DSC, TGA, and DTG analyses. The DSC thermogram of pristine LAA, demonstrated an endothermic peak at 190 °C, implying this to be its melting point (Figure 4a) [42]. Comparatively, the DSC thermogram of HP-*β*-CD demonstrated a plane DSC curve, due to its amorphous nature. The observed DSC thermogram of LAA/HP-*β*-CD–NF resembled the pristine HP-*β*-CD thermogram. Moreover, no endothermic peak of LAA was observed in the LAA/HP-*β*-CD-NF thermogram, revealing that the LAA was efficiently incorporated in the cavity of HP-*β*-CD.

The TGA graphs of pristine LAA, HP-*β*-CD, and LAA/HP-*β*-CD-NF are presented in (Figure 4b). The LAA started decomposing at around ~190 °C, and, at around ~220 °C the rate of decomposition reached a maximum [43]. Additional decomposition was observed when increasing the temperature stepwise, from 240 to 400 °C. Approximately 20% of the initial sample was left, as a charred residue, at 700 °C [44]. The thermal heating of pristine HP-*β*-CD demonstrated two different phases of weight loss, due to dehydration and deterioration. The dehydration of the samples causes a weight loss from 30 to 100 °C and degradation arises between 310 and 390 °C [28]. For LAA/HP-*β*-CD-NF, a different TGA pattern was seen; there are three main phases of weight loss, attributed to the dehydration, and deterioration of LAA and HP-*β*-CD, respectively. Due to water evaporation, LAA/HP-*β*-CD-NF demonstrated a first weight loss step below 120 °C (similar to what was observed in pristine HP-*β*-CD). The LAA was degraded between 240 and 310 °C, followed by a phase of the thermal breakdown of HP-*β*-CD, from 310–370 °C [25]. It was observed in thee TGA thermogram, as well as in the derivative curves, that the thermal degradation of LAA in LAA/HP-*β*-CD-NF was transferred to a higher temperature, of about 350 °C, above the degradation temperature of HP-*β*-CD (~330 °C) (Figure 4c). Moreover, it was also noticed that the main degradation step of LAA (190–240 °C) was reduced and shifted to a slightly higher temperature range in LAA/HP-*β*-CD-NF (220–310 °C), confirming the inclusion of LAA inside the HP-*β*-CD, these results agree with previously published reports [45,46]. For example, the incorporation of ferulic acid in the HP-*β*-CD cavity, led to an improvement in the thermal stability of the ferulic acid, by enhancing its thermal degradation temperature [25]. Moreover, Narayan et al. reported that the thermal stability of active molecules improves after their inclusion in CD cavities [47]. According to the current study, LAA’s thermal stability was found to be increased when it was enclosed inside the HP-*β*-CD cavity. Moreover, HP-*β*-CD can stabilize LAA, by raising its thermal degradation temperature.

### 3.7. Nuclear Magnetic Resonance (NMR) Studies

Nuclear magnetic resonance (NMR) spectroscopy can be used to investigate the intermolecular interactions between the guest and host assemblies, by comparing the chemical shifts in the NMR spectrum before and after the formation of the inclusion complex [48]. The formation of the inclusion complex, causes a slight shifting in protons (downfield/upfield) of the guest and host molecules that are in close proximity [49]. Therefore, we used ^1^H NMR to compare the chemical shift changes between LAA, HP-*β*-CD, and LAA/HP-*β*-CD-NF, in order to determine the formation of the inclusion complex.

**Figure 5:**
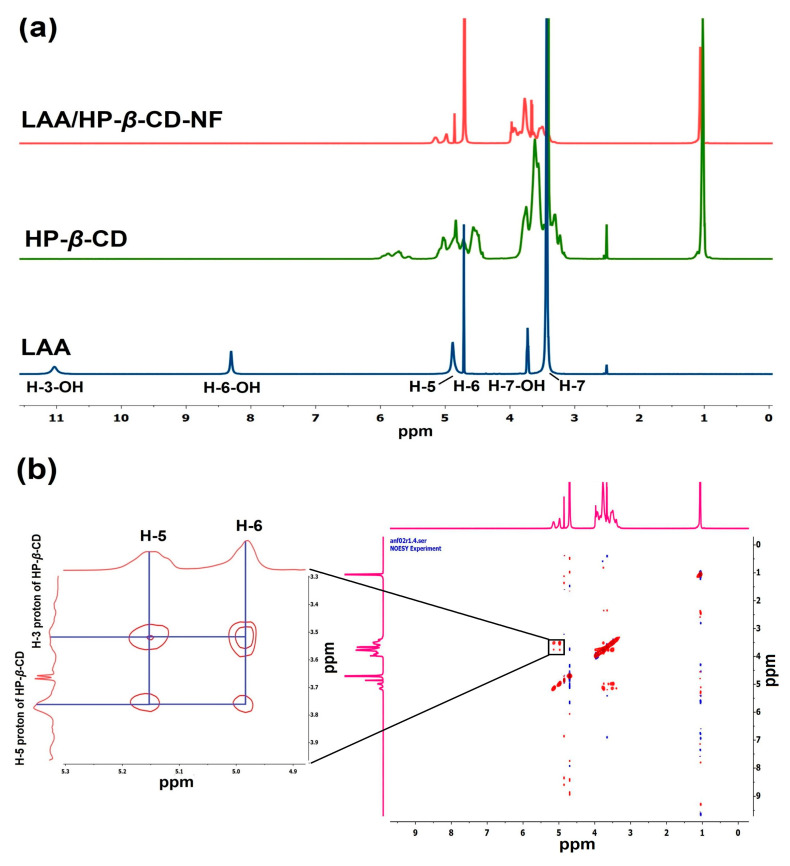
Nuclear magnetic resonance spectroscopy characterization. (**a**) ^1^H NMR of LAA, HP-*β*-CD in DMSO-d_6_, and LAA/HP-*β*-CD-NF in D_2_O, (**b**) 2-D NOESY spectrum of LAA/HP-*β*-CD-NF in D_2_O, demonstrating contour region (zoomed in region), revealing interaction of LAA protons with protons of HP-*β*-CD.

The proton NMR spectrum of LAA has peaks at ~11.02 ppm, ~5.77 ppm, and at ~3.72 ppm due to –OH groups of H-3-OH, H-6-OH, and H-7-OH, respectively. Additionally, peaks at ~4.88 and ~4.71 ppm were observed, due to 5-CH ring proton(s) and 6-CH proton(s), respectively. Furthermore, a peak at 3.46 ppm was observed due to 7-CH2 proton(s) (Figure 5a) [50]. The chemical shifts of HP-*β*-CD were found at values of ~4.83 ppm, ~3.47 ppm, ~3.75 ppm, ~3.41 ppm, ~3.56 ppm, ~3.61 ppm, and at ~1.02 ppm, attributable to H-1, H-2, H-3, H-4, H-5, and H-6 protons, and methyl group protons, respectively [40,51]. 

A comparative assessment of the proton spectra of LAA and LAA/HP-*β*-CD-NF was conducted, in order to verify the incorporation of LAA inside the HP-*β*-CD cavities. It was clearly observed in the NMR spectrum of LAA/HP-*β*-CD-NF, that the H-5 and H-6 protons of LAA showed considerable upfield (∆δ), of 0.0107 ppm and 0.0273 ppm, respectively (Supplementary Table S2) (calculated using the equation (∆δ = δ LAA/HP-*β*-CD-NF − δ LAA)).

To gain a thorough understanding of the mechanisms of inclusion of the guest inside the host cavities, we also used the 2D-NMR approach. The nuclear Overhauser effect (NOE), is a common phenomenon observed in NMR spectroscopy of inclusion complexes, through transfer of spin polarization from one population to another (host–guest), occurring between the atoms in close proximity to each other [49]. Any two protons that are located within 0.4 nm in space, can produce a nuclear Overhauser effect cross-correlation, in NOE spectroscopy (NOESY) [11]. The correlation of NOE cross peaks to their respective molecular distances, provides more detailed information about the interaction of protons of the guest–host assembly, making data interpretation simple [10,40]. Thus, we further performed 2D-NOESY analysis, to understand the molecular interactions behind the inclusion of LAA inside the HP-*β*-CD cavities.

Typically, it has been found that after the formation of an inclusion complex, the CD protons (H-3 and H-5) present inside the cavity experience a rise in electron density, that changes their chemical shifts, but the protons outside the cavity (H-1, H-2, and H-4) are unaffected. The 2D-NOESY spectrum of LAA/HP-*β*-CD-NF, demonstrated that there was a significant correlation between the 5-CH ring proton and 6-CH proton of LAA, with the H-3 and H-5 protons of HP-*β*-CD (Figure 5b). The correlation of LAA with HP-*β*-CD causes induced shifting of the H-3 and H-5 protons of HP-*β*-CD present in the inner cavity (H-3 (∆δ = 0.026 ppm) and H-5 (∆δ = 0.074) ppm). These small shifts suggest interaction of LAA with HP-*β*-CD protons (Supplementary Table S2). All these findings demonstrated the possible inclusion of LAA inside the HP-β-CD cavity. Similar observations were reported by Saha et al.: two different vitamins (nicotinic acid and ascorbic acid) were encapsulated inside the *β*-CD cavity, and a 2D-NMR experiment demonstrated appreciable correlation of the protons of the studied vitamins with the H-3 and H-5 protons of β-CD [11].

### 3.8. DPPH Antioxidant Radical Scavenging Activity

**Figure 6:**
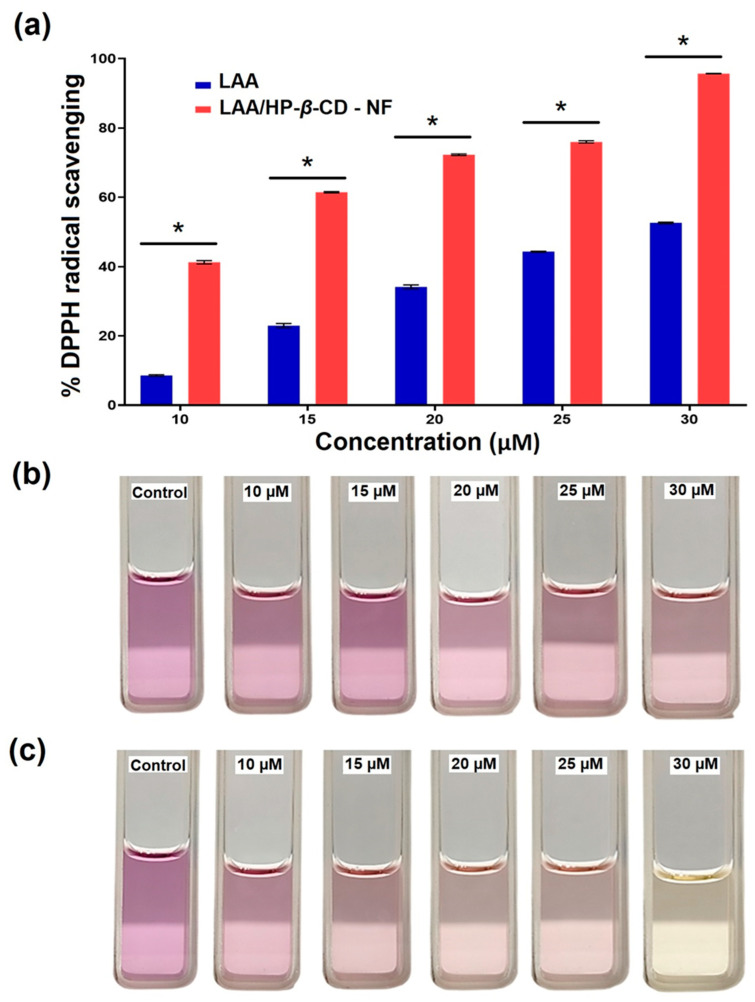
(**a**) Graphical representation of concentration-dependent antioxidant performance (% DPPH radical scavenging) of LAA and LAA/HP-*β*-CD-NF. Visual representation of series of different concentrations of obtained solutions of (**b**) LAA and (**c**) LAA/HP-*β*-CD-NF, demonstrating change in color of sample solutions (due to scavenging of DPPH radical), from purple to yellow at highest concentration of LAA in LAA/HP-*β*-CD-NF (30 µM), indicating its antioxidant potential. The values are expressed as mean ± standard deviation (n = 3), and the statistical analysis at the significance level of * *p* < 0.05 was achieved by using one-way analysis of variance (ANOVA), followed by Tukey’s post hoc test, using GraphPad Prism 9 software.

Reactive oxygen species (ROS), or free radicals, can oxidize a variety of biomolecules (including DNA, proteins, and lipids), and are known to be the root cause of cancer, stroke, and neurodegenerative diseases [52]. Antioxidant agents can hinder the destructive consequence of free radicals and ROS, by scavenging them. Here, the DPPH assay was used to compare the antioxidant potential of LAA with LAA/HP-*β*-CD-NF; a reduction in the optical density of DPPH (at 517 nm) was recorded. At the highest LAA concentration (30 µM), LAA/HP-*β*-CD-NF demonstrated 95.66 ± 0.07% radical scavenging (Figure 6a). In contrast, the pure LAA showed the highest (52.61 ± 0.19%) radical scavenging at 30 µM. These results were further supported by the DPPH assay images, which illustrate that the color of the solution of LAA/HP-*β*-CD-NF turned yellow, whereas the comparative LAA sample solution had a light purple hue (Figure 6b). When compared to pure LAA, LAA/HP-*β*-CD-NF has a much higher antioxidant capacity. This is because LAA in LAA/HP-*β*-CD-NF is more thermally stable, due to its amorphous state and the inclusion of LAA in HP-*β*-CD cavities, that protects it from oxidation. Similar observations have been recorded, where vitamin E/HP-*β*-CD-NF demonstrated enhanced antioxidant potential as compared to free vitamin E [20]. These findings demonstrated that complexation of LAA with HP-*β*-CD efficiently enhances the antioxidant potential of LAA, due to protection of LAA inside the CD cavities, and enhanced the solubility of LAA, due to its conversion into an amorphous NFs state.

## 4. Conclusions

LAA serves as an effective free radical scavenger in pharmaceutical and food products. Multiple factors restrict the therapeutic and food application of LAA, such as its instability, rapid degradation, and poor bioavailability. In recent years, cyclodextrins have gained enormous attention in the pharmaceutical and food sectors, as a means of enhancing the poor solubility, instability, and bioavailability of various active molecules, by forming inclusion complexes. Moreover, cyclodextrin inclusion-incorporated active molecules provide a polymer-free electrospinning solution to developing electrospun fibers, and as such, have attracted a lot of attention in scientific studies and the industrial sector. In the current research work, the polymer-free inclusion complex-based NF sheets of LAA were fabricated using modified *β*-CD derivative hydroxypropyl–*β*–cyclodextrin (HP-*β*-CD). The continuous variation method (Job plot) demonstrated a 2:1 molar guest–host stoichiometric ratio. The free standing, defect free, and homogeneous LAA/HP-*β*-CD-NF were obtained via optimized parameters of the electrospinning technique, with an average diameter of ~339 nm. The ultimate NFs demonstrated efficient LAA entrapment, with entrapment efficiency of 97.30%. The solid-state characterizations (PXRD, FT-IR, and ^1^H NMR and 2D-NOESY) reveal effective evidence of the inclusion of LAA within the HP-*β*-CD cavity. The thermal behavioral studies showed that the main degradation step of LAA (190–240 °C) has been reduced and shifted to a slightly higher temperature range in LAA/HP-*β*-CD-NF (220–310 °C), revealing the formation of the inclusion complex and conversion into NFs, that led to the enhanced thermal stability of LAA. Moreover, a DPPH radical scavenging assay demonstrated that LAA/HP-*β*-CD-NF exhibits enhanced antioxidant potential compared to LAA.

It is worth stating that, the electrospinning process was carried out using water as a solvent, with a polymer-free electrospinning solution, offering a beneficial advantage for the industrial scale commercialization of the LAA-incorporated inclusion complex NFs. Moreover, the developed LAA/HP-β-CD-NF can be incorporated as excipients in a variety of products, such as food packaging material, facial masks, and wound healing dressings [15,16,22,23]. Cyclodextrin inclusion complex-loaded NFs not only provide avenues to surmount limitations and curbs associated with LAA, but can also broaden the therapeutic and food application of LAA.

## Data Availability

The data is included in the article.

## Supplementary Materials

The following supporting information can be downloaded at: https://www.mdpi.com/article/10.3390/foods12071363/s1. The supplementary information includes Figure S1: HPLC overlay chromatogram of various concentration of L-ascorbic acid (LAA), Table S1: Data for the continuous variation method (Job’s plot) carried out using UV-Visible spectrophotometer for aqueous LAA/HP-β-CD system and Table S2: 1H NMR chemical shifts (δ, ppm) and chemical shift differences (∆δ, ppm) of free LAA and HP-β-CD inclusion complexes.
