# Supplementary material for: Preparation, Characterization, and Antioxidant Activity of L-Ascorbic Acid/HP-β-Cyclodextrin Inclusion Complex-Incorporated Electrospun Nanofibers
Source: Foods. 2023 Mar 23;12(7):1363. doi: 10.3390/foods12071363 (PMC10093489; doi:10.3390/foods12071363)
Supplement: Supplementary file 1 [file foods-12-01363-s001.zip › foods-2292235-supplementary.pdf]

Preparation, Characterization and Antioxidant Activity of L-Ascorbic Acid/ HP-*β*-Cyclodextrin Inclusion Complex Incorporated Electrospun Nanofibers

Nabab Khan[a,b], Amit Kumar Singh[a,b], Ankit Saneja[a,b]

[a]Formulation Laboratory, Dietetics and Nutrition Technology Division, CSIR-Institute of Himalayan Bioresource Technology, Palampur-176061, Himachal Pradesh, India

[b]Academy of Scientific and Innovative Research (AcSIR), Ghaziabad-201002, Uttar Pradesh, India

***Corresponding Author**

Dr. Ankit Saneja
Scientist
Dietetics and Nutrition Technology Division,
CSIR – Institute of Himalayan Bioresource Technology,
Palampur,176061,
Himachal Pradesh, India
E-mail: ankitsaneja@ihbt.res.in ankitsaneja.ihbt@gmail.com
Tel:91-1894-233339; Ext: 485

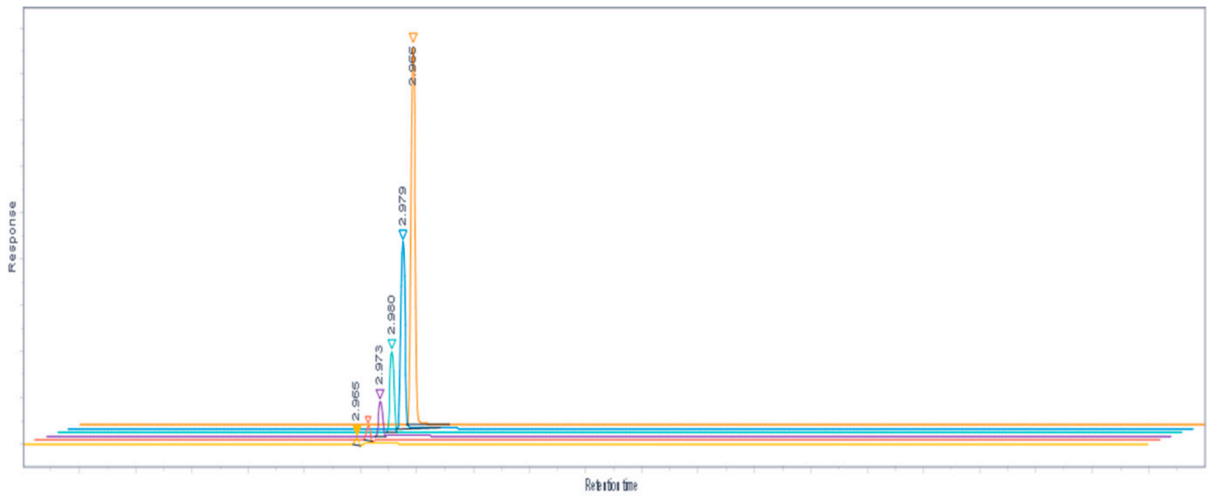

Figure S1: HPLC overlay chromatogram of various concentration of L-ascorbic acid (LAA)

Table S1: Data for the continuous variation method (Job's plot) carried out using UV-Visible spectrophotometer for aqueous LAA/HP-*β*-CD system.

LAA (mL)	HP-*β*-CD (mL)	LAA (μM)	HP-*β*-CD (μM)	[(LAA)/ (LAA) + (HP-*β*-CD)]	Absorbance (A)	∆A	∆A × [(LAA)/ (LAA) + (HP-*β*-CD)]
0.0	1.0	0	100	0.0	0.000	0.0000	0.0000
0.1	0.9	10	90	0.1	0.152	0.1298	0.0129
0.2	0.8	20	80	0.2	0.307	0.2238	0.0447
0.3	0.7	30	70	0.3	0.434	0.2953	0.0886
0.4	0.6	40	60	0.4	0.535	0.3045	0.1218
0.5	0.5	50	50	0.5	0.569	0.2682	0.1341
0.6	0.4	60	40	0.6	0.617	0.2435	0.1461
0.7	0.3	70	30	0.7	0.720	0.1747	0.1223
0.8	0.2	80	20	0.8	0.774	0.1351	0.1081
0.9	0.1	90	10	0.9	0.870	0.0431	0.0388
1.0	0.0	100	0	1.0	0.995	0.0000	0.0000

Table S2: ^{1}H NMR chemical shifts (δ, ppm) and chemical shift differences (Δδ, ppm) of free LAA and HP-*β*-CD inclusion complexes

**Protons of LAA**	**($\delta_{free\ LAA}$)**	**($\delta_{complex}$) with HP-*β*-CD**	**($\Delta\delta = \delta_{complex - free\ LAA}$) with HP-*β*-CD**
H-3-OH	11.02	n.r	-
H-6-OH	5.77	n.r	-
H-7-OH	3.72	n.r	-
H-5	4.88	4.85	0.0273
H-6	4.71	4.70	0.0107
CH2	3.46	n.r	-
**Protons of HP-*β*-CD**			
H-1	4.836	n.r	-
H-2	3.474	n.r	-
H-3	3.747	3.773	0.026
H-4	3.412	3.410	0.002
H-5	3.562	3.636	0.074
H-6	3.614	3.637	0.023
CH3	1.023	1.062	0.039

* n.r represents peaks that are not resolved in the spectrum after complexation
